# Exploring the Link Between Causative Agents of Healthcare-Associated Infections and Predisposing Factors Causing Extended Hospital Stays

**DOI:** 10.7759/cureus.94309

**Published:** 2025-10-10

**Authors:** Ahmed Felifel, Amir Soltan, Mohamed Emad Abolseoud, Huda Elsayed Eltotongy, Fares Elnagar, Mohamed Ebid, Fayrouz El Shawadfy, Mohamed Eldamaty, Amr Abouelezz, Esraa Saber, Mostafa Afifi, Hussein Hany Hussein Saber Ghonaim, Ahmed Younis Elashmawy, Mosaab Altayar, Amjad Algharaibeh, Mohab Elsalahi, Salma Hisham Ahmed Ali Mohamed, Zahraa Mohamed Mostafa Abousobh, Aya Sayed, Sara Osama Eldeib, Abdulmabod Omar

**Affiliations:** 1 Department of Emergency Medicine, Barnet Hospital, Royal Free London NHS Foundation Trust, London, GBR; 2 Faculty of Medicine, Alexandria University, Alexandria, EGY; 3 Department of Internal Medicine, Kafrelsheikh University Hospital, Kafr El Sheikh, EGY; 4 Department of Cardiology, 6 October Insurance Hospital, Giza, EGY; 5 Faculty of Medicine, Mansoura University, Mansoura, EGY; 6 Faculty of Medicine, Tanta University, Tanta, EGY; 7 Department of Surgery, Badr Hospital, Cairo, EGY; 8 Faculty of Medicine, Zagazig University, Zagazig, EGY; 9 Faculty of Medicine, Al-Azhar University, Cairo, EGY; 10 Department of Cardiology, Royal Papworth Hospital, NHS Foundation Trust, Cambridge, GBR; 11 Department of General Surgery, NHS Lanarkshire, Glasgow, GBR; 12 Faculty of Medicine, Alexandria Univeristy, Alexandria, EGY; 13 Faculty of Medicine, Ain Shams University, Cairo, EGY; 14 Department of Laboratory, Dr. Hassan Ghazzawi Hospital, Abeer Medical Group, Jeddah, SAU

**Keywords:** cohort study, hospital hygiene, hospital stay, klebsiella pneumoniae, multidrug-resistant pathogens, nosocomial infection, predisposing factors

## Abstract

Background

Healthcare-related infections initially meant those infections that developed during a stay in an extended-care hospital, but currently are used to describe the infections that develop in the continuum of healthcare settings where individuals receive care.

Aim

The aim of this study is to explore the relationship between causative agents of healthcare-associated infections (HAIs) and predisposing factors contributing to extended hospital stays. It seeks to generate insights that improve patient care, optimize outcomes, and reduce infection-related burdens.

Method

This retrospective cohort study was conducted at Dr. Hassan Ghazzawi Hospital, Jeddah, Saudi Arabia, using data from the medical records department. All patients who received an HAI diagnosis while in the hospital between January 2024 and December 2024 made up the research population. In line with this background, this study investigated the distribution of causative agents of infection in hospitalized patients (n = 60) with respect to gender and length of hospital stay. The study design and reporting followed the Strengthening the Reporting of Observational Studies in Epidemiology (STROBE) guidelines.

Results

Data were collected on 10 major pathogens, including *Klebsiella pneumoniae*, *Acinetobacter baumannii*, and methicillin-resistant *Staphylococcus aureus* (MRSA), among others. The Kruskal-Wallis test was applied to assess differences in mean hospital stay, while the Chi-square test was utilized to assess the relation between gender and hospital-born causative agents. Result shows that gender differences were not statistically significant for any of the infections (all p > 0.05). An independent t-test of the total pathogen counts in males and females revealed no significant difference (t-test p = 0.594). The F-test, which compares variance, also revealed no discernible variation in group variability (F = 0.964, p = 0.521). Although certain infections, such as *Klebsiella pneumoniae* and *Acinetobacter baumannii*, were more frequent, the overall distribution did not differ significantly by gender. The Kruskal-Wallis test was conducted to determine whether hospital stay differed significantly across patients with different causative agents of HCAIs. Statistical analysis did not demonstrate a significant difference in hospital stay between the different causative agents. One-way analysis of variance (ANOVA) yielded F = 1.65 and p = 0.126, and the nonparametric Kruskal-Wallis H test also confirmed nonsignificance (H = 7.96, p = 0.538). This indicates that the type of causative agent was associated with variations in the length of hospital stay, meaning variation in hospital stay appears to be more patient-specific rather than strongly dependent on the causative agent of infection.

Conclusion

These findings suggest that factors other than pathogen type and gender such as severity of illness, underlying comorbidities, host immune status, treatment strategies, timeliness of intervention, antimicrobial resistance (AMR) patterns, hospital hygiene regulation, adequacy of infection control practices, staffing ratios, availability of critical care resources, and overall quality of the hospital care system may have a greater influence on patient outcomes.

## Introduction

The evidence of the existence and inappropriate consequences of healthcare-associated infections (HAIs) has long been established in the literature over the past few decades. HAIs initially meant those infections that developed during a stay in an extended-care hospital (previously known as a nosocomial infection), but currently are used to describe the infections that develop in the continuum of healthcare settings where individuals receive care (e.g., long-term care, home care, outpatient care) [1]. Such unexpected infections occur during healthcare treatment processes and lead to serious illnesses in patients (which can be morbidity and mortality), extended length of hospital stay, and require further diagnostic and treatment procedures. All of these developing conditions add monetary expenses to patients despite the existing costs incurred by the underlying ailment, which is why HAIs are viewed as a negative consequence, because they may affect the quality of patient care, cause adverse events, and pose a patient safety concern.

Similarly, the World Health Organization (WHO) classified it as a disease that did not exist or was incubating at the time of hospitalization but that appeared 48 hours or longer after admission [2]. According to the WHO estimates, HAIs affect hundreds of millions of patients each year, with developing nations bearing a disproportionate amount of the burden because of their negligent surveillance, limited antimicrobial stewardship programs, and inadequate infection control measures [3].

Furthermore, the rise in antimicrobial resistance (AMR) further compounds the burden of HAIs, as resistant pathogens are increasingly implicated in these infections, limiting treatment options and threatening progress in modern medicine [4]. Recent literature indicates that organisms such as *Klebsiella pneumoniae*, *Acinetobacter baumannii*, *Candida auris*, *Clostridium difficile*, *Enterococcus faecium*, methicillin-resistant *Staphylococcus aureus* (MRSA), vancomycin-resistant *Enterococci *(VRE), *Pseudomonas aeruginosa*, *Escherichia coli*, and *Proteus mirabilis* are among the leading causative agents of HAIs worldwide [5]. Each of these pathogens is associated with unique virulence mechanisms and high resistance rates, complicating infection control and therapeutic strategies [6].

Parallel to AMR, equally important are the predisposing factors that render hospitalized patients more vulnerable to HAIs. These include prolonged hospital stay, invasive procedures such as central venous catheters and endotracheal intubation, underlying comorbidities (e.g., diabetes mellitus, renal disease, ischemic heart disease, cerebral hemorrhage, and hypertensive encephalopathy), immunosuppression, burns, surgical interventions, and many more. Conditions such as septic shock, hepatic coma, disseminated intravascular coagulopathy, and diabetic ketoacidosis further impair immune responses, thereby facilitating colonization and infection by opportunistic pathogens [7]. These clinical risk factors highlight the complexity of preventing HAIs, as they are not solely the result of pathogen exposure but arise at the intersection of microbial virulence, patient vulnerability, and healthcare practices.

Despite the extensive recognition of HAIs as a global problem, there is limited cohort-based data in many healthcare systems, especially in low- and middle-income countries. Most available studies are cross-sectional or retrospective, which restricts understanding of temporal relationships between risk factors, infection onset, and clinical outcomes. A cohort design is particularly valuable because it allows systematic follow-up of hospitalized patients, enabling the identification of incident cases, establishing causality between predisposing factors and infection risk, and assessing the impact of specific pathogens on patient outcomes such as length of hospital stay and mortality. Consequently, the current cohort study is planned to investigate the causative agents and predisposing factors of hospital-acquired infections within the hospital setting. By systematically identifying the microbial spectrum and the clinical conditions that increase susceptibility to HAIs, this study aims to generate evidence that can inform infection control strategies, antimicrobial stewardship, and policy interventions tailored to the local healthcare context. The findings are expected to strengthen surveillance, guide empirical therapy, and ultimately reduce the burden of HAIs on patients, clinicians, and the healthcare system at large.

## Materials and methods

Research design

A retrospective cohort study approach was used in this investigation, as it involved reviewing previously collected medical records of hospitalized patients with confirmed HAIs. The study design and reporting followed the Strengthening the Reporting of Observational Studies in Epidemiology (STROBE) guidelines [8].

Research setting

The research was conducted at Dr. Hassan Ghazzawi Hospital, Jeddah, Saudi Arabia, using data from the medical records department. The hospital admits a wide range of patients with medical, surgical, and intensive care needs, making it a suitable setting for studying HAIs and related factors.

Ethical approval

The research protocol was approved by the Institutional Review Board of Dr. Hassan Ghazzawi Hospital, Jeddah, Saudi Arabia, with IRB approval number DHGH-MBEC-2404 on June 10, 2025. The study was conducted in accordance with the principles of the Declaration of Helsinki [9].

Research population

All patients who received an HAI diagnosis while in the hospital between January 2024 and December 2024 made up the research population. The inclusion criteria of selected candidates in this study are as follows: (a) patients of all ages and both sexes with confirmed HAIs, (b) cases with microbiologically verified causative organisms, and (c) patients whose medical records included complete information on demographics, predisposing factors, causative organisms, and hospital stay. The exclusion criteria of selected candidates in this study are as follows: (a) patients with community-acquired infections, (b) cases with incomplete or missing records, and (c) patients discharged within 48 hours (to exclude colonization rather than infection).

Data collection

Retrospective data extraction was done from microbiological lab results and hospital records. The variables collected included the following: (a) demographic data, i.e., gender, age; (b) clinical data, i.e., type of infection, causative agent, and presence of predisposing factors (e.g., cerebral hemorrhage, burns, diabetes, indwelling devices, immunosuppression); and (c) outcome variables, i.e., hospital stay duration in days.

Data were organized into tables (e.g., distribution of causative organisms by gender, association with predisposing factors, and mean length of hospital stay). The following independent variables (causative agents, predisposing factors) and dependent variables (occurrence of infection, hospital stay duration) are carefully taken into consideration during this study.

Statistical analysis

IBM SPSS Statistics for Windows, Version 25 (Released 2017; IBM Corp., Armonk, New York, United States) was used to analyze the data curation, and MS Excel (Microsoft Corporation, Redmond, Washington, United States) was used for presentation. For continuous variables (hospital stay length), mean ± standard deviation was employed, whereas frequencies and percentages were utilized for categorical variables (e.g., causative agent, gender, predisposing factors). The relationship between predisposing variables and infection type, as well as between gender and causative agent type, was evaluated using the Chi-square test. However, because hospital stay data were not normally distributed, the Kruskal-Wallis test was used as a nonparametric method to compare median hospital stay lengths among various pathogenic species. A p-value of less than 0.05 was deemed statistically significant during the work.

## Results

For this cohort study, a total of 60 patients were included after following all the selection criteria (Table 1). The study population comprised 26 males (43.3%) and 34 females (56.7%). The mean age for the selected patients was found to be approximately 52 years, ranging from 18 to 88 years, demonstrating that the cohort included both young adults and elderly individuals. The average duration of hospital stay of the patient was found to be 17 days, with stays ranging from two to 88 days, suggesting substantial variability in clinical course and disease severity. Furthermore, nearly two-fifths of the patients required mechanical ventilation, while the remaining 37 patients were managed without ventilatory support. This distribution reflects the heterogeneity of disease severity and underlying conditions within the study cohort.

**Table 1:** Demographics and HAIs patients’ characteristics HAIs: healthcare-associated infections

Category	Value
Total patients (n)	60
Male (n, %)	26 (43.3%)
Female (n, %)	34 (56.7%)
Mean age (years)	~52
Age range (years)	18-88
Mean hospital stay (days)	~17
Hospital stay range (days)	2-88
Mechanical ventilation: yes (n, %)	23 (38.3%)
Mechanical ventilation: no (n, %)	37 (61.7%)

Table 2 describes that there is no statistically significant association between gender (male/female) and the type of causative agent. It was found that two Gram-negative bacteria, i.e., *Klebsiella pneumoniae* and *Acinetobacter baumannii, *were the most opportunistic pathogens to cause longer hospital stay in patients. The prevalence of pathogens like* Proteus mirabilis *and *Escherichia coli *was comparatively low in both groups. To evaluate the distribution of each pathogen between male and female patients, Chi-square tests were used. It was revealed that gender differences were not statistically significant for any of the infections (all p > 0.05). An independent t-test of the total pathogen counts in males and females revealed no significant difference (t-test p = 0.594). The F-test, which compares variance, also revealed no discernible variation in group variability (F = 0.964, p = 0.521).

**Table 2:** Association between gender and distribution of causative agents *t-test (p = 0.595), f-test (0.964, p = 0.521); **NS = not significant

Pathogen	Male	Female	Mean	SD	Chi-square (p-values)	Significance
Klebsiella pneumoniae	6	8	7	1.41	1.000	NS
Acinetobacter baumannii	8	4	6	2.83	0.173	
Candida albicans	3	4	3.5	0.71	1.000	
Clostridium difficile	1	5	3	2.83	0.299	
Enterococcus faecium	3	3	3	0	1.000	
Methicillin-resistant *Staphylococcus aureus*	1	5	3	2.83	0.299	
Pseudomonas aeruginosa	1	3	2	1.41	0.755	
Vancomycin-resistant *Enterococci*	2	0	1	1.41	0.386	
Escherichia coli	1	1	1	0	1.000	
Proteus mirabilis	1	0	0.5	0.71	0.919	

Table 3 describes the relation between causative agents and hospital stay in patients. The data reveal that Gram-negative bacteria, particularly *Klebsiella pneumoniae *and *Acinetobacter baumannii*, were the predominant causative agents of hospital-acquired infections, suggesting a substantial burden on patient recovery and healthcare resources. Among 60 patients with hospital-acquired infections demonstrated that *Klebsiella pneumoniae *was the most frequently isolated pathogen, accounting for 14 cases, with a mean hospital stay of approximately 19 days.* Acinetobacter baumannii *was the second most common, identified in 12 patients, with an average stay of 15 days. Interestingly, while VRE infections were rare (3.3%), they were linked to the longest average hospital stay (~20 days), highlighting their clinical impact despite low prevalence. Conversely,* E. coli* accounted for only 3.3% of cases and was associated with the shortest hospital stay (~9 days), suggesting relatively less severe outcomes in this sample.

**Table 3:** Distribution of causative agents and mean hospital stay in ICU patients (n = 60) MRSA: methicillin-resistant *Staphylococcus aureus*; VRE: vancomycin-resistant *Enterococci*; ANOVA: analysis of variance One-way ANOVA (F-test): F = 1.65, p = 0.126 → NS;  Kruskal-Wallis H test: H = 7.96, p = 0.538 → NS; **NS = not significant

Causative agent	n	Mean stay (days)	SD
Klebsiella pneumoniae	14	35.71	44.93
Acinetobacter baumannii	12	31.08	36.54
Candida albicans	7	47.29	46.83
Clostridium difficile	6	26.5	12.41
Enterococcus faecium	6	16.5	13.49
MRSA	6	32.17	28.24
Pseudomonas aeruginosa	4	53	84.67
VRE	2	134	65.05
Escherichia coli	2	20.5	17.68
Proteus mirabilis	1	12	—

However, the mean hospitalized stay varied widely among different pathogens, ranging from 12 days for *Proteus mirabilis* to 134 days for VRE. Considerable variability in length of stay was observed within groups, with the largest standard deviations noted for *Klebsiella pneumoniae *(SD = 44.93), *Candida albicans *(SD = 46.83), and *Pseudomonas aeruginosa* (SD = 84.67). Statistical analysis did not demonstrate a significant difference in hospital stay between the different causative agents. One-way ANOVA yielded F = 1.65, p = 0.126, and the nonparametric Kruskal-Wallis H test also confirmed nonsignificance (H = 7.96, p = 0.538). This indicates that the type of causative agent was associated with variations in the length of hospital stay. In other words, the variation in hospital stay appears to be more patient-specific rather than strongly dependent on the causative agent of infection. At the same time, we cannot ignore the fact that although the mean hospital stay appeared longer in patients infected with VRE and *Pseudomonas aeruginosa*, these differences were not statistically significant when compared with other pathogens. The lack of significance is likely attributable to the relatively small sample size in certain groups (e.g., *Proteus mirabilis*, n = 1; VRE, n = 2; *Escherichia coli*, n = 2), as well as the high variability in hospital stay across patients. These findings suggest that while some pathogens may be associated with prolonged hospital stays, the current dataset does not provide sufficient evidence for a statistically significant effect. Larger and more evenly distributed cohorts are needed to clarify whether these trends are clinically meaningful.

**Figure 1:**
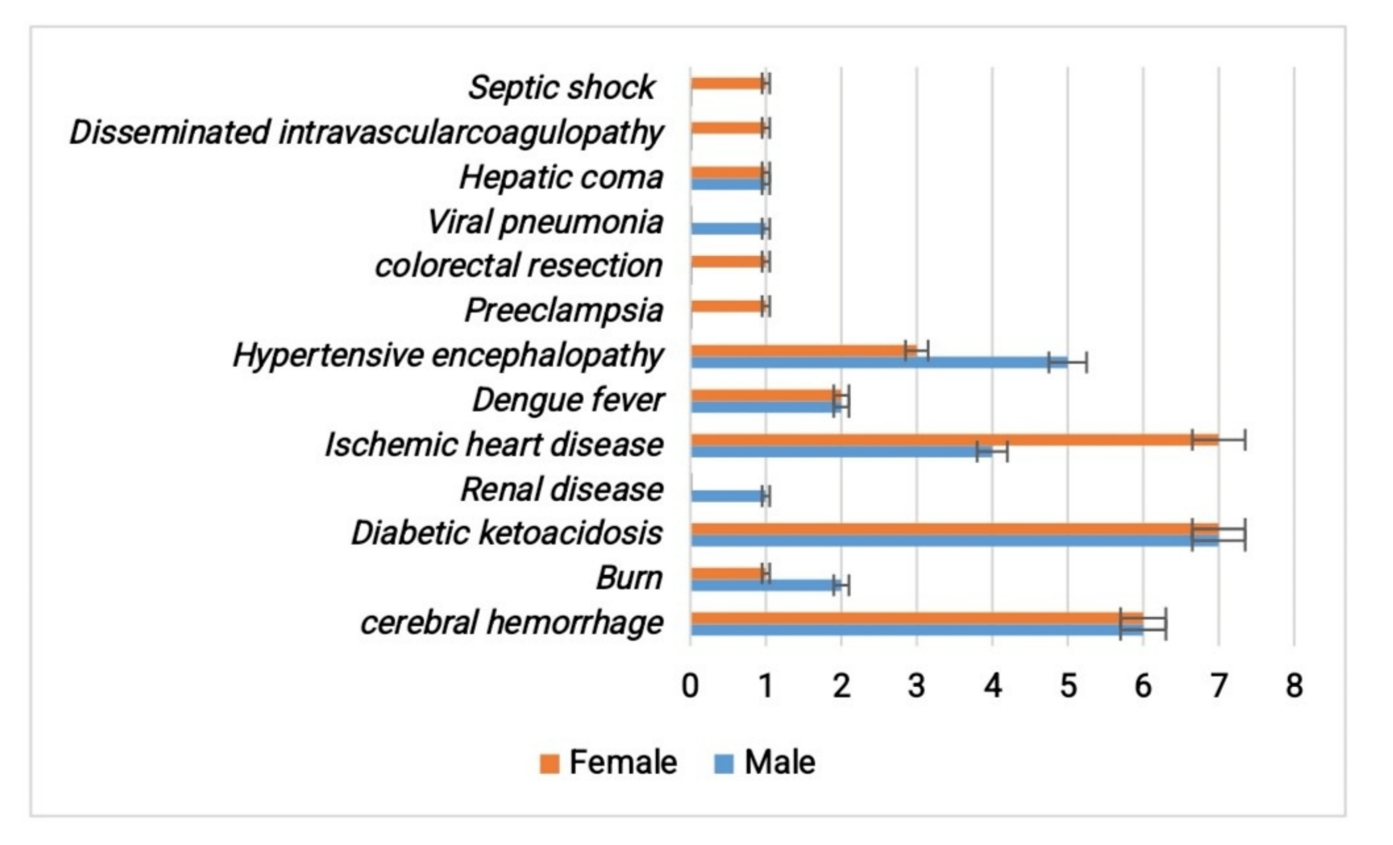
Predisposing factors that require the patient to stay in the hospital

The analysis of predisposing factors among patients whose hospital stay was prolonged due to secondary infections with multidrug-resistant organisms. Most common predisposing conditions were diabetic ketoacidosis (14 cases; 7 males, 7 females), cerebral hemorrhage (12 cases; 6 males, 6 females), and ischemic heart disease (11 cases; 4 males, 7 females). Other conditions, such as hypertensive encephalopathy (8 cases) and burn injuries (3 cases), were also observed. Rare predisposing factors were also observed (Figure 1). Overall, no strong sex-specific predisposition was noted.

## Discussion

This cohort study assessed the demographic characteristics, predisposing factors, causative agents, and outcomes among critically ill patients admitted to the hospital. The findings provide valuable insights into the epidemiology of hospital-acquired infections and their association with clinical outcomes such as length of stay and need for mechanical ventilation. The study revealed that females (56.7%) were slightly more represented than males (43.3%), with a mean patient age of 52 years, indicating that middle-aged and older populations are particularly vulnerable. These findings align with previous studies that reported advanced age as a significant risk factor for hospital (ICU) infections due to weakened immunity and frequent comorbidities [10,11]. The mean hospital stays of 17 days, with some patients requiring prolonged admission up to 88 days, highlight the burden of nosocomial infections on healthcare resources.

The analysis of predisposing conditions demonstrated a wide spectrum, with diabetic ketoacidosis, ischemic heart disease, hypertensive encephalopathy, and cerebral hemorrhage being among the most common. These conditions are associated with systemic immune suppression and metabolic instability, predisposing patients to infection and adverse outcomes. Similar patterns were reported in previous cohorts, where metabolic and cardiovascular comorbidities were strongly correlated with infection risk and ICU mortality [12,13]. More precisely, studies conducted in hospitals have demonstrated that diabetic patients have a markedly increased risk of ICU-acquired bloodstream infections; even when their blood sugar was closely monitored, their risk of bloodstream infections was 1.7 times higher than that of nondiabetics (hazard ratio = 1.66; 95% CI, 1.04-2.64; p = 0.034) [14]. According to a nationwide analysis, individuals with underlying cardiovascular problems who contracted even one HAI saw an absolute 8.9% increase in in-hospital mortality, 6.6 more hospital days, and more than double the cost of healthcare [15]. The identification of these factors in our study reinforces the need for early risk stratification and targeted preventive interventions in vulnerable patients. Among the isolated pathogens, *Klebsiella pneumoniae* and* Acinetobacter baumannii*, the predominance of Gram-negative organisms as nosocomial agents is consistent with global hospital ICU observation studies [16,17], where multidrug-resistant bacteria remain a major threat to patient safety. Our findings are in line with international data showing an increasing prevalence of multidrug-resistant Gram-negative organisms in ICUs, especially in the case of *Klebsiella pneumoniae*, which most common nosocomial infection found in hospitalized patients [16,18]. Additionally, a worldwide survey found that 28.69% of *Klebsiella pneumoniae *infections were caused by carbapenem-resistant *Klebsiella pneumoniae* (CRKP), with South Asia having the highest frequency (66.04%) [19]. The fact that *Klebsiella pneumoniae* strains isolated from intensive care unit patients were resistant to carbapenems and other antibiotics, underscoring the concerning proliferation of resistant strains in hospital environments, is what further exacerbates the problem [20,21]. However, compared with developed countries, the relatively higher presence of* Acinetobacter baumannii *in our study may reflect regional AMR patterns and resource limitations in infection control [22,23]. This emphasizes the importance of local epidemiological surveillance to guide empirical treatment decisions. 

Moreover, the proportion of patients requiring mechanical ventilation (38.3%) indicates the severity of illness in this cohort and underscores the risk of ventilator-associated pneumonia, as reported before [24,25]. Prolonged ICU stays and invasive interventions likely facilitated colonization and infection by multidrug-resistant organisms. These results emphasize how crucial stringent infection control procedures, antimicrobial stewardship, and early mobilization tactics are in reducing the incidence of bloodstream infections and VAP.

Limitations

This study has limitations stemming from its relatively small sample size (n = 60) under observation. Being a single-center study, the findings may not be representative of the larger population or different hospital settings within the same region. Additionally, the microbiological data were limited to culture-positive cases, which could lead to an underestimation of the prevalence of infections. Finally, the analysis of patient outcomes, such as mortality, was not conducted in depth, which limits the assessment of long-term effects.

## Conclusions

The study demonstrates that neither gender nor type of causative agent showed a statistically significant impact on hospital stay in this cohort. While some pathogens were more common, their distribution was similar across males and females. However, predisposing variables such as underlying comorbidities (e.g., diabetes, cardiovascular disease, and renal diseases), prior antibiotic exposure, and the use of invasive devices like ventilators and catheters are likely to have an impact on outcomes. These factors may be more important in determining the severity and duration of hospitalization than the pathogen type itself. This highlights the need for broader investigations into patient-specific and clinical factors influencing outcomes, alongside continued efforts to control the spread of resistant pathogens. To reduce nosocomial infections and improve patient care and outcomes, it is recommended that the hospital prioritize strict infection prevention measures such as regular hand hygiene audits, thorough environmental cleaning, and adherence to aseptic standards during invasive procedures. To prevent AMR, empirical antibiotic therapy should be guided by local resistance patterns and antibiograms, as well as timely de-escalation methods. Early detection and management of patients with predisposing diseases, such as diabetes, renal illness, and cardiovascular disorders, can aid in reducing consequences and promoting targeted prevention. Careful monitoring and the most effective use of invasive devices, as well as evidence-based ventilatory procedures including subglottic secretion drainage and early weaning, can help to prevent infection-related morbidity. Strengthening staff training, promoting interdisciplinary care coordination, and adopting continuous surveillance systems are all critical to improving patient safety and clinical results. Larger multicenter studies in the same region healthcare center are recommended to validate these results and assess long-term outcomes, including mortality and quality of life.
